# Total intestinal atresia with failure of recanalization extending from the duodenum to the rectum: The first case report

**DOI:** 10.1016/j.ijscr.2024.110668

**Published:** 2024-11-30

**Authors:** Raghad Samha, Hossam Tharwat Ali, Layal Msheik, Tia Khadra, Aamer Zainab

**Affiliations:** a Faculty of Medicine, AlBaath University, Homs, Syrian Arab Republic; b Qena Faculty of Medicine, South Valley University, Qena, Egypt; c Faculty of Medical Sciences, Lebanese University, Beirut, Lebanon; d Medical Learning Skills Academy, Beirut, Lebanon; e Department of Pediatrics, Almadina Hospital, Damascus, Syrian Arab Republic

**Keywords:** Atresia, Duodenum, Obstruction, Laparotomy, Total atresia

## Abstract

**Introduction and importance:**

Intestinal atresia is a congenital condition characterized by the blockage or constriction of an intestine portion. It is caused by either a vascular issue or a failure of recanalization during fetal development. Surgery is essential for both a definitive diagnosis and therapy.

**Case presentation:**

A preterm neonate (gestational age 34 weeks) was delivered by cesarean section due to polyhydramnios and complained of recurrent episodes of non-bilious vomiting after breastfeeding. Prenatal ultrasound revealed a double bubble sign, indicating intestinal atresia, while on contrast imaging, the contrast was refluxed from the stomach to the esophagus, the absence of gases in the intestine or colon suggested a gastric outlet obstruction. The atretic intestine was identified during laparotomy, from the second section of the duodenum to the rectum with no lumen. There were no surgical options available. Atresia is typically treated by excision and anastomosis of the remaining normal canalized intestine. However, in our case, a total intestinal atresia was discovered, and no intestinal lumen was identified to undergo surgery.

**Clinical discussion:**

It was found that the atretic intestine in our case extended from the duodenum to the rectum. However, in the two previously documented cases of atresia, the atresia began in the jejunum and progressed to the rectum in one instance and the sigmoid colon in the other.

**Conclusion:**

Because there were no alternative surgical options, the intestine was relocated, and the abdomen was closed. Such cases require more sensitive diagnostic instruments to recognize, and probable etiologies should be researched further to prevent them.

## Highlights

• Most cases have small intestinal atresia whereas colonic atresia is rare.
• Most cases have isolated atresia in one part of the intestine with the duodenum accounting for nearly half of cases.
• Our case is the first published case with total intestinal atresia

## 1 Introduction

Intestinal atresia is a congenital disorder marked by the absence, narrowing, or obstruction of a section of the intestines [1]. It is one of the most prevalent causes of intestinal blockage, affecting one out of every 5000 babies. Atresia can develop in a variety of places, including the duodenum (50 % of cases) and the jejunum and ileum (39 % of cases) [2]. Two basic explanations have been presented for its genesis. According to Tandler's Concept, intestinal atresia is caused by a lack of recanalization during the early stages of intestinal development. However, according to Louw and Barnard's research, atresia is caused by vascular accidents during fetal development [3]. In infants, intestinal atresia manifests as severe vomiting, abdominal distension, inability to pass meconium, reduced bowel motions, feeding problems, dehydration, and probable weight loss [1,3]. Prenatal ultrasonography was found to be more effective in identifying duodenal atresia than distal atresia, increasing the rate of prenatal diagnosis [3,4]. Intestinal atresia is diagnosed by clinical examination, imaging (X-rays, ultrasound), and maybe contrast investigations to reveal intestinal obstruction. Surgery is frequently required for a clear diagnosis, with the location and type of obstruction verified during the surgery [5]. Intestinal atresia is treated surgically by removing the blocked or constricted section of the intestines and creating an anastomosis between the two unaffected ends. The nature and location of the atresia determine the surgical method [1]. In this paper, we present a case of a premature neonate with entire intestinal atresia, where no intestinal lumen was found during exploratory laparotomy. This work has been reported in line with the SCARE criteria [6].

## 2 Case presentation

A preterm neonate (gestational age 34 weeks, birthweight 2700 g) delivered by cesarean section due to polyhydramnios to a primigravida 25-year-old mother, presented to the Pediatrics department complaining of recurrent episodes of non-bilious vomiting following breastfeeding.

**Fig. 1:**
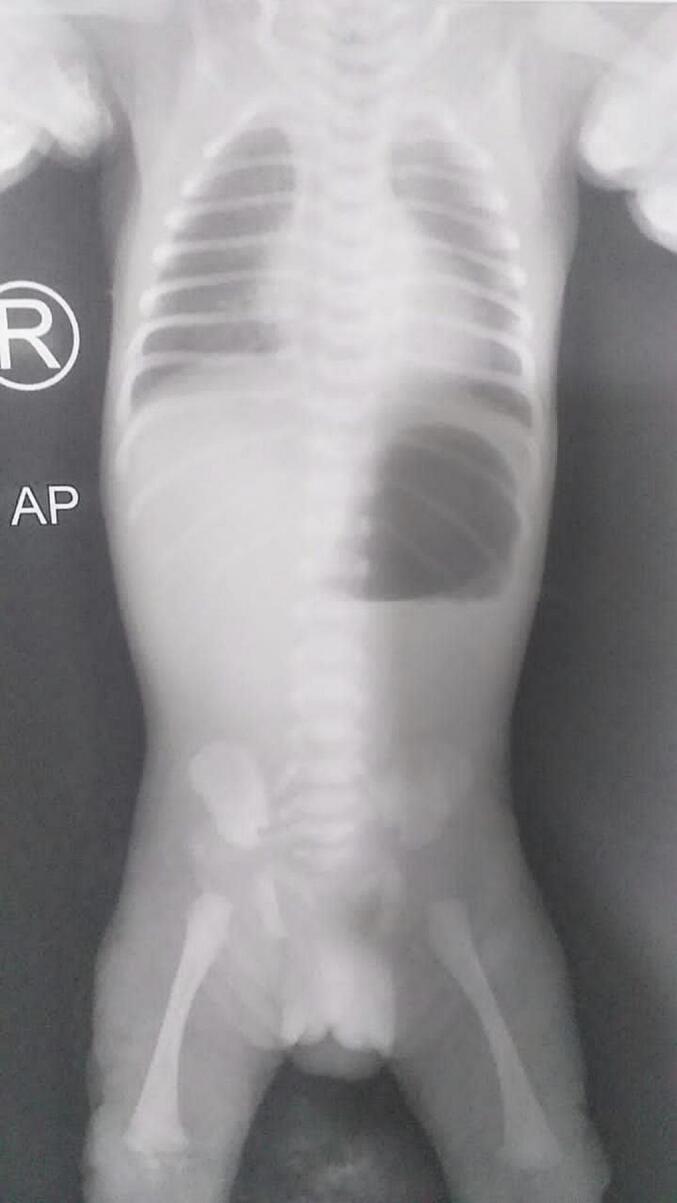
A plain abdominal X-ray demonstrated a huge, dilated stomach.

**Fig. 2:**
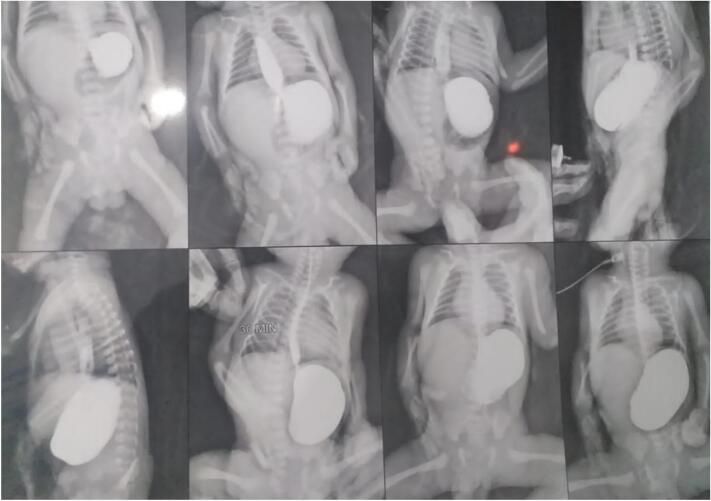
contrast imaging showed a stomach obstruction, with the frequent reflux of contrast agent into the esophagus, and the absence of gases in the intestines and colon, suggesting a Gastric outlet obstruction (GOO).

Prenatal Ultrasound (US) demonstrated polyhydramnios and suggested duodenal atresia. There was no significant contributing family history. On clinical examination, the abdomen was soft and not distended, external anal orifice was normal, respiratory, and hemodynamic status was stable, and there were no obvious dysmorphic features. Vital signs were within normal limits. A plain abdominal X-ray showed a huge, dilated stomach (Fig. 1). On contrast imaging, stomach obstruction was found, noting the frequent reflux of contrast agent into the esophagus, along with the absence of gases in the intestines and colon which suggested a Gastric outlet obstruction (GOO)(Fig. 2).

**Fig. 3:**
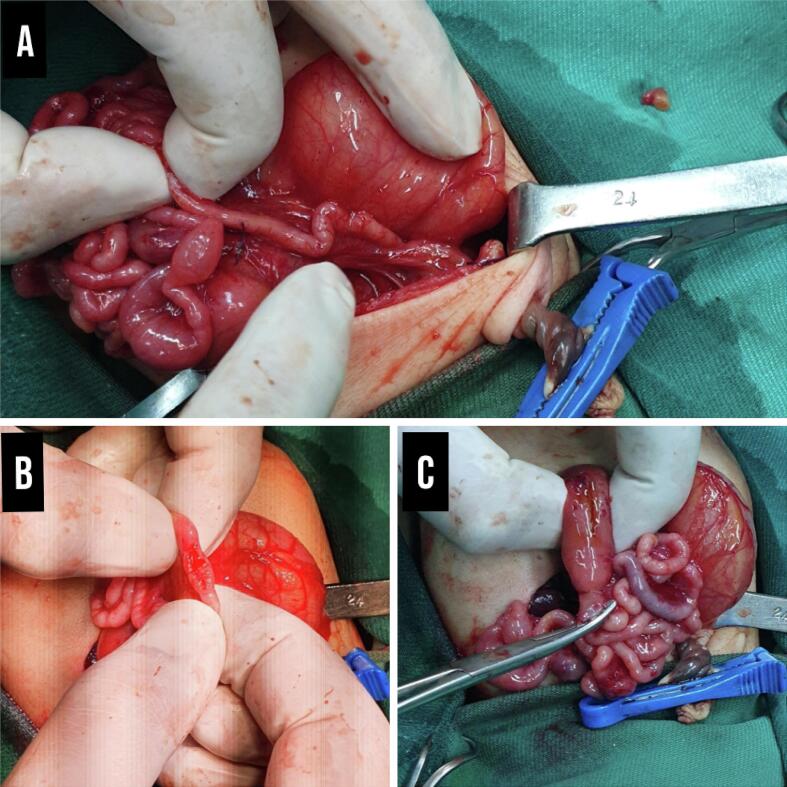
(A, B, and C): A: the atresia extending to the end of the colons. B: multiple enterotomies from the site of the atresia to the end of the colons were performed and confirmed no lumen. C: dilated stomach and the first and second duodenal segments, with second duodenal segment atresia.

An exploratory laparotomy was performed using a supraumbilical transverse incision. The stomach and the first and second segments of the duodenum were found to be severely dilated. The second duodenal segment was atretic. Additionally, the bile duct was dilated, and the gallbladder was enlarged due to the associated duodenal atresia and impaired bile drainage. A small intestinal cyst was also identified, likely caused by the accumulation of mucus secretions. The remainder of the intestine was examined for continuity after the duodenum's atretic section. Unexpectedly, multiple enterotomies from the site of the atresia to the end of the colons confirmed that no lumen was found in intra-testing from the duodenum to the rectum (Fig. 3). What was left of the duodenum, jejunum, ileum, and Colon was a single solid fibrous cord extending the entire length of the small and large intestines (video 1). There was no available surgical option, enterotomy closure was made by multiple sutures, the intestines were relocated back, and the abdomen was closed.

After receiving parenteral nutrition for 72 h, the patient died.

## 3 Discussion

Intestinal obstruction constitutes one of the most common emergency conditions among neonates [7]. Early diagnosis and surgical interventions are the cornerstone of intestinal salvage and hence better prognosis. Additionally, serious complications that can be fatal as ischemic bowel, aspiration pneumonia, or failure of nutrition require well-equipped healthcare facilities with organized multidisciplinary teams [8]. Mortality rates may reach half of the cases in developing countries such as African countries, probably due to late diagnosis and limited resources [8].

Intestinal atresia is the major cause of intestinal obstruction in neonates, accounting for more than one-third of the cases with an approximate incidence of 1 in 5000 neonates [7]. It is noteworthy that most cases have small intestinal atresia whereas colonic atresia is quite rarer. Most cases have isolated atresia in one part of the intestine with the duodenum accounting for nearly half of cases while ileum and jejunum are affected in around 39 % of cases [2]. Very few cases [[10], [11], [12], [13]] were reported to have multiple segment atresia while only two [[7], [8], [9], [10], [11], [12], [13], [14]] were reported to have near-total jejuno-ileal atresia with no distal lumen (Table 1).

**Table 1:** Reported cases of total intestinal atresia.

Reference	Gender	Family history	Obstetric history	General condition of the newborn	Level of the atresia	Mesentery	Other congenital anomalies; anal opening	Surgical management	Fate of the newborn
Aggerwal et al., 2019	Male	None	NR	Stable; no signs of sepsis or syndromic changes	Jejunum (type IIIa) to rectum	Mesenteric defects only in part of jejunum.	No other anomalies with normal anal opening.	Exploratory laparotomy: end duodenostomy and reposition of the intestine.	NR (The parents took home two days post-operative)
Morris-Stiff et al., 1998	NR	NR	Pre-term labor at 36 weeks.	Healthy with no visible abnormalities	Proximal jejunum to distal sigmoid colon	NR	Gastroschisis diagnosed antenatally, no evident at delivery.	Laparotomy: no option was decided and hence closed.	Died a few days later.
The present case	Male	None	Polyhydramnios; C-section at 34 weeks	Well; no signs of sepsis or syndromic changes	Duodenum to rectum	NR	No other anomalies with normal anal opening.	Laparotomy: no option was decided and hence closed.	Died three days after the surgery.

NR: No Record.

Table legend: A literature review of total intestinal atresia cases, which includes two cases starting from the jejunum, and our presented case starting from the duodenum.

Our case was found to have an atretic intestine from the duodenum to the rectum. On the other hand, the previous two cases of atresia started at the jejunum and to the sigmoid colon in one case [14] and to the rectum in the other [7].

So far, the exact underlying pathology of intestinal atresia is not understood. Vascular compromise that leads to ischemic changes is hypothesized especially in the ileo-jejunal atresia cases that are associated with other bowel abnormalities in the form of gastroschisis, intussusception, and volvulus [8]. Chromosomal abnormalities such as Down syndrome are present in nearly 40 % of duodenal atresia cases while they are rare with ileo-jejunal cases [15].

Prenatal ultrasound allows earlier diagnosis and better prognosis, especially in cases with duodenal atresia [15]. On the other hand, in ileo-jejunal cases, prenatal ultrasound findings can suggest intestinal obstruction in only 29–50 % of cases [16]. Thus, most cases are diagnosed after birth and more accurately upon surgical exploration. The defects in parts of the mesentery were detected in the case reported by Aggerwal *et al* [7] while no changes were detected in our case. Overall, intestinal atresia has favorable outcomes despite the misdiagnosis upon prenatal ultrasound in most typical cases [9].

The usual surgical management of typical cases of intestinal atresia is resection and anastomosis of the remaining normal canalized viable part of the intestine [8,15,16]. Sham *et al* presented a case of near-total atresia involving multiple ileo-jejunal segments. The child had a total length of remaining small intestine of less than 10 cm with a normal colon that allowed the performance of multiple anastomoses [17]. Herein all three cases, since the atresia involved almost all small and large intestines, there were not enough remaining small or large intestines to perform resection and anastomosis. In our case, except for the first and second parts of the duodenum, the small and large intestinal tracts were a single fibrous cord. Hence, no intraoperative option was decided for all cases, and they were closed after repositioning the intestine. Two of the three cases died days post-operative while the third was discharged home with parents and lost for follow-up.

## 4 Conclusion

In conclusion, raising awareness of such anomalies can help with early detection, proper management, and collaborative efforts targeted at enhancing both palliative care practices and prospective advances in whole intestine atresia therapy alternatives. Given the complexity of treatment and management, it is imperative for pediatric surgeons to carefully evaluate and determine an appropriate approach for patients with total intestinal atresia to achieve optimal outcomes.

The following is the supplementary data related to this article.

Supplementary video 1 A video that was recorded during the surgery, shows the whole intestine without a lumen. Supplementary video 1

## Abbreviations list

US: Ultrasound
GOO: Gastric outlet obstruction

## CRediT authorship contribution statement

R.S: design of the study, data collection, data interpretation and analysis, drafting, critical revision, approval of the final manuscript.

H.T.A: data interpretation, and analysis, critical revision, drafting, approval of the final manuscript.

L.M: drafting, critical revision, approval of the final manuscript.

T.K: drafting, critical revision, approval of the final manuscript.

A.Z: The Supervisor, patient care, drafting, critical revision, approval of the final manuscript.

## Ethical approval

Not required for this case report.

## Guarantor

Dr. Aamer Zainab is the guarantor of this work.

## Research registration number

NA.

## Consent for publication

Written informed consent was obtained from parents of the patient for publishing this case report and any accompanying images. A copy of the written consent is available for review by the Editor-in-Chief of this journal.

## Sources of funding

No funding was required.

## Declaration of competing interest

The authors declare that they have no conflicts of interest.
